# The impact of adenoid hypertrophy across pediatric age groups on maxillomandibular development and position

**DOI:** 10.1016/j.jped.2026.101524

**Published:** 2026-03-05

**Authors:** Xue-Ru Zhang, Jian-Ya Zhang

**Affiliations:** Department of Otorhinolaryngology, Children’s Hospital of Nanjing Medical University, Nanjing, Jiangsu, China

**Keywords:** Adenoid hypertrophy, Craniofacial development, Mandibular retrusion, Dental malocclusion, Pediatric airway obstruction

## Abstract

**Objective:**

Although adenoid hypertrophy (AH) is associated with malocclusion, its age-specific impact on craniofacial development across different dentition stages remains unclear. This study aimed to assess AH-related craniofacial changes across distinct phases of dental development.

**Methods:**

This retrospective study analyzed 180 children divided into three age groups: 3–5, 5–8, and 8–11 years. Lateral cephalometric radiographs were used to evaluate sagittal skeletal relationships (SNA, SNB, ANB), dentoalveolar position (NA-Apo), occlusal plane inclination (NPo–FH), jaw lengths (ANS-Ptm, Co-Gn), and overjet. Comparisons were made between the AH and control groups within each age cohort.

**Results:**

In the 3–5 year group, no significant craniofacial differences were found between AH and control subjects. Between 5–8 years, AH was linked to a significantly lower SNB angle and higher NA-Apo angle (p < 0.05). By 8–11 years, AH patients showed significantly reduced SNA, SNB, and NPo–FH angles, along with increased ANB, NA-Apo, and overjet values compared to controls (p < 0.05).

**Conclusion:**

AH was associated with age-dependent craniofacial changes—such as mandibular retrusion, dental protrusion, posterior occlusal tilt, and increased overjet—without altering jaw length. Early diagnosis and timely intervention during key growth stages are crucial to prevent long-term facial abnormalities. Given the retrospective, 2D cephalometric design, causality could not be inferred, and longitudinal or treatment effects could not be assessed.

## Introduction

Adenoid hypertrophy (AH) is a common upper airway obstruction in children, with prevalence rates ranging from 42 % to 70 %, highlighting its public health significance [1]. Clinically, AH commonly presents with nasal obstruction, snoring, and mouth breathing, which can lead to obstructive sleep apnea, chronic hypoxia, and associated issues such as poor sleep, recurrent infections, and cognitive impairment [2]. More importantly, AH is also linked to craniofacial abnormalities and an increased risk of malocclusion, which is more prevalent in affected children than in healthy peers [3]. Because upper-airway obstruction and craniofacial growth vary across childhood, the dentofacial impact of AH may differ by developmental stage. Therefore, this study aimed to characterize dentition-stage–specific craniofacial changes associated with AH across three pediatric age groups (3–5, 5–8, and 8–11 years).

Extensive evidence shows that AH disrupts nasal breathing and significantly affects craniofacial growth [1,4]. The primary mechanism is chronic mouth breathing, which promotes mandibular retrusion and limits maxillary growth, producing the characteristic “adenoid facies” with increased lower facial height and an altered mandibular angle [5]. Cephalometrics in children with AH often reveal reduced SNB, increased ANB, and a steeper MP/SN angle, consistent with vertical growth and a skeletal Class II pattern [[6], [7], [8]]. AH may also induce dentoalveolar compensation and occlusal adaptation, contributing to clinically relevant malocclusion features [7].

The influence of AH on craniofacial morphology appears to be age-dependent, and both onset timing and symptom duration have been associated with the magnitude of dentofacial alterations [6,9]. Thus, dentition stage–based analyses are needed to determine when AH-related skeletal and dentoalveolar changes become detectable [6,7,9].

Despite growing evidence linking AH to craniofacial abnormalities, age-stratified analyses of related skeletal and dentoalveolar changes across dentition stages remain limited [6,7,9]. Many studies combine wide pediatric age ranges and/or assess only a few cephalometric variables, which may mask stage-specific patterns [6,10]. Therefore, participants were stratified by dentition stage (3–5, 5–8 and 8–11 years), and an integrated panel of skeletal (SNA, SNB, ANB), dentoalveolar (NA–Apo), functional/occlusal (NPo–FH) and clinical (overjet) indices was evaluated to determine when adenoidal hypertrophy–associated dentofacial changes become detectable and clinically relevant. This age-stage–specific approach is intended to inform the timing of airway assessment and individualized orthodontic planning.

## Materials and methods

### Participants

This retrospective study included 180 children who underwent lateral cephalometric radiography. Written consent was obtained from the guardians. Participants were divided into three age groups based on dentofacial development stages: 3–5 years (deciduous dentition), 5–8 years (early mixed dentition), and 8–11 years (late mixed to early permanent dentition). Stratified sampling was performed by age group, and participants within each stratum were selected using a random number table. Each group consisted of 30 children with AH and 30 age-, sex-, and BMI-matched controls.

AH was diagnosed based on clinical symptoms and radiographic evidence of upper airway obstruction. Control subjects had no history of airway obstruction, sleep-disordered breathing, craniofacial anomalies, or orthodontic treatment. Patients were recruited from otolaryngology and pediatric dentistry clinics, while controls were selected from children receiving routine dental or health evaluations.

### Inclusion criteria

This retrospective analysis included children with available lateral cephalograms who met the following criteria: 3–11 years of age; pre-pubertal skeletal maturity (CS1 or CS2) determined by the cervical vertebral maturation method; a high-quality pretreatment lateral cephalogram; radiographic evidence of adenoidal and/or tonsillar hypertrophy; no history of adenoidectomy or tonsillectomy; and no prior orthodontic treatment.

### Exclusion criteria

Exclusion criteria were as follows: (1) a history of tonsillectomy, adenoidectomy, or previous orthodontic treatment; (2) severe skeletal malocclusions necessitating surgical intervention; and (3) craniofacial trauma or diagnosed syndromic anomalies.

### Cephalometric analysis

All participants underwent standardized lateral cephalometric radiography before treatment using a digital X-ray system (OC200, Instrumentarium Dental, Finland). Radiographs were obtained with subjects in an upright position, ensuring the Frankfort horizontal plane was parallel to the floor. Patients were instructed to maintain centric occlusion and refrain from movement, speaking, or swallowing during image acquisition. Lateral cephalometric radiographs were jointly acquired and preliminary screened by two experienced radiologists to ensure that image quality met diagnostic criteria. Maxillomandibular parameters were independently measured by three orthodontists using Huazheng digital cephalometric analysis software, with a blinded assessment approach to minimize subjective bias.

**Fig. 1:**
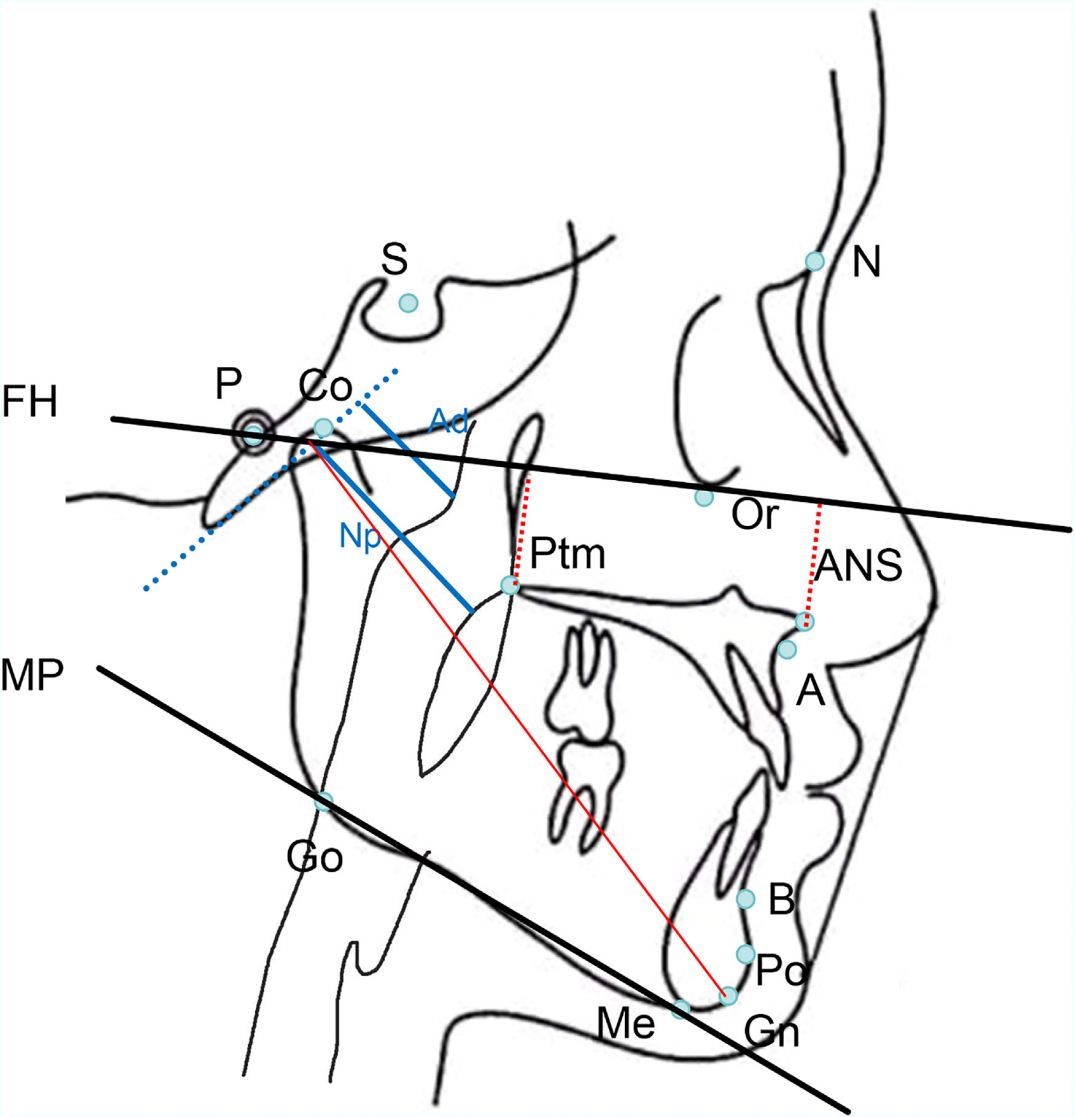
Angular and linear measurements of the cephalometric analysis. Sagittal jaw relationships.

Cephalometric landmarks and measurements in this study are shown in Figure 1. The cephalometric analysis identified key anatomical landmarks, including sella (S), nasion (N), point A (subspinale), point B (supramentale), menton (Me), gonion (Go), and gnathion (Gn). Reference planes included the Frankfort horizontal plane (orbitale–porion) and the mandibular plane (menton-gonion). Angular and linear measurements assessed were as follows: SNA (sella–nasion–point A angle), SNB (sella–nasion–point B angle), and ANB (point A–nasion–point B angle), which reflect the anteroposterior relationship of the maxilla and mandible; FH—NPo (Frankfort horizontal to occlusal plane angle) and NPo-FH (mandibular plane to Frankfort horizontal plane angle), indicative of vertical jaw relationships; ANS-Ptm (distance from the anterior nasal spine to the pterygomaxillary fissure), and Co-Gn (linear distance from the condylion to the gnathion), representing maxillary depth and mandibular length, respectively. The position of the adenoid (Ad) and the posterior border of the nasopharynx (Np) were used to calculate the adenoidal size (Ad/Np), while tonsillar size was evaluated using the ratio of tonsillar width to oropharyngeal width (Tn/Op). A ratio greater than 0.5 was considered indicative of hypertrophy in both regions.

### Concordance analysis

For intra-operator reliability, each orthodontist repeated cephalometric measurements in 20 % of randomly selected records after a 2-week washout period. For inter-operator reliability, measurements obtained independently by the three orthodontists on the same records were compared. Reliability was quantified using the intraclass correlation coefficient (ICC), with ICC ≥ 0.75 indicating good agreement. Following confirmation of acceptable reliability, the final values used for analysis were calculated as the mean of the three examiners’ measurements.

### Sample size calculation

Based on previous evidence, the minimum required sample size per group was estimated to be 30 using G*Power (Heinrich Heine University Düsseldorf), assuming a large effect size (Cohen’s *d* = 0.8), a two-sided significance level of α = 0.05, and 90 % power (1−β = 0.90).

### Statistical analysis

Potential confounding was mitigated through age-stratified sampling and by matching controls to AH cases on age, sex and BMI within each stratum. Residual clinical heterogeneity was further limited by restricting analyses to prepubertal skeletal maturity (CS1–CS2) and by applying prespecified exclusion criteria, including prior orthodontic treatment, adenotonsillectomy, severe skeletal malocclusion, craniofacial trauma and syndromic craniofacial anomalies.

Continuous variables were presented as mean ± standard deviation (SD), and categorical data as frequencies and percentages [n ( %)]. Between-group comparisons were performed using independent-samples *t*-tests for continuous variables and chi-square tests for categorical variables. During data analysis, we compared the values of SNA, SNB, ANB, NA-Apo, NPo-FH, ANS-Ptm, Co-Gn, and overjet between girls and boys of AH patients within each age group. No statistically significant differences were detected, and thus no further sex adjustment was performed. A p-value < 0.05 was considered statistically significant.

## Results

### The included participants for analysis

**Fig. 2:**
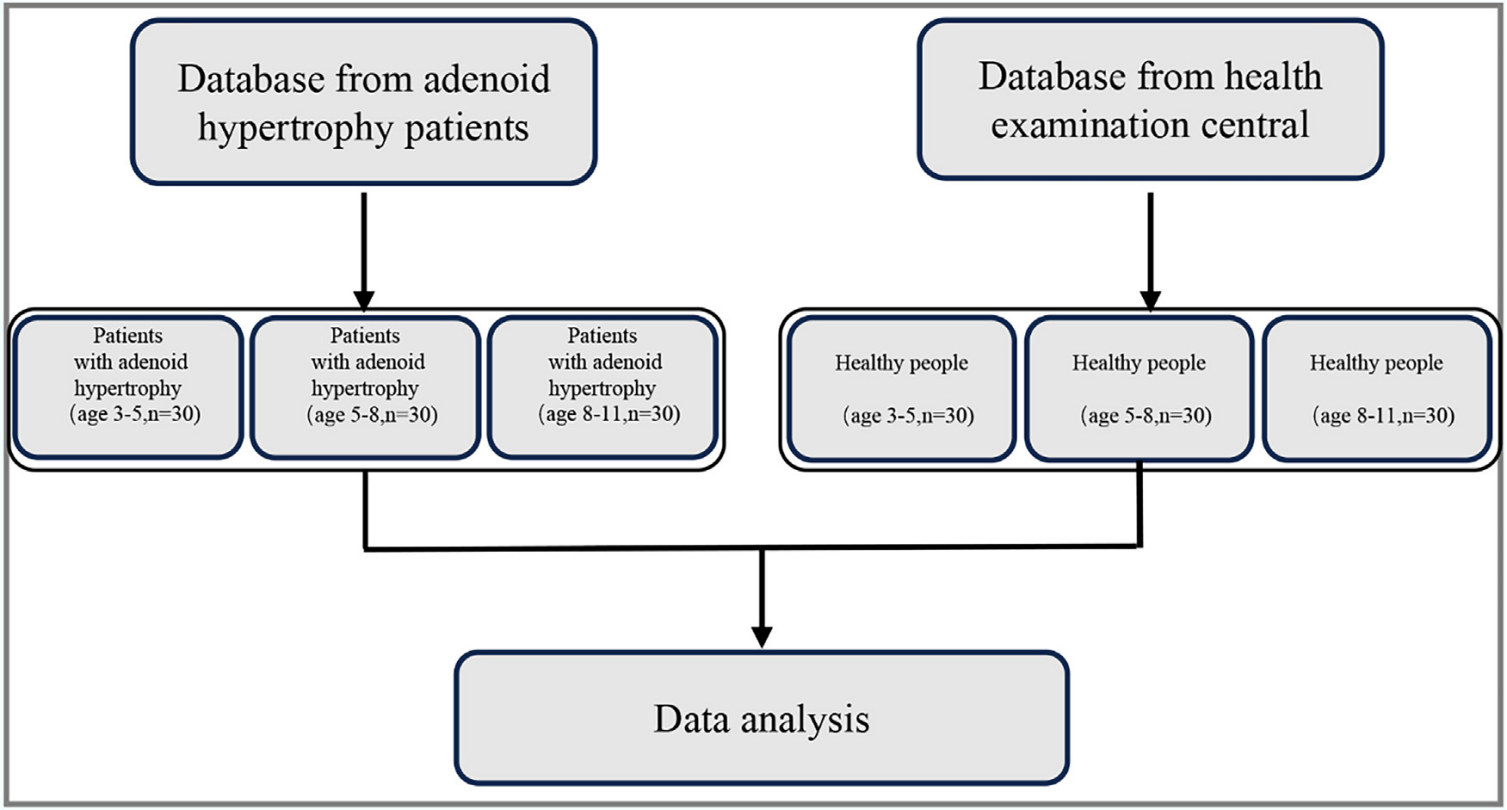
Flow chart of the study participants. SNA: sella-nasion-A point angle; SNB: sella-nasion-B point angle; ANB: A point-nasion-B point angle; FH—NPo: Frankfort horizontal-occlusal plane angle; NPo-FH: Mandibular plane to Frankfort horizontal plane angle; ANS-Ptm: anterior nasal spine- pterygomaxillary fissure; Co-Gn: the point of the condyle to gnathion; Ad: adenoidal point; Np: nasopharynx posterior.

A total of 180 participants were included in the analysis, stratified into three age groups: 3–5 years, 5–8 years, and 8–11 years. Each age group comprised 30 patients diagnosed with adenoid hypertrophy and 30 age-matched healthy controls (Figure 2). AH cases were drawn from a clinical database, and controls were recruited from a health examination center (Figure 2). All six subgroups were analyzed to evaluate age-specific craniofacial and physiological differences associated with AH.

### Sociodemographic characteristics

To ensure the validity of comparisons between groups, we assessed whether the AH and control groups were demographically comparable across three age cohorts (3–5, 5–8, and 8–11 years). As shown in Table 1, no significant differences were observed in chronological age between groups in any age stratum (*p* = 0.108, 0.065, and 0.086, respectively), indicating successful age matching. Gender distribution was balanced across all groups (*p* > 0.4), and BMI values were comparable, with no significant differences detected (*p* = 0.662, 0.563, and 0.744, respectively), suggesting similar growth and nutritional status. Parental education levels (illiterate, primary, secondary, graduate or above) did not differ significantly between AH and control groups in any cohort (*p* = 0.757, 0.235, and 0.190), indicating similar sociodemographic characteristics. Similarly, no differences were observed in area of residence (urban vs. rural) across age groups (*p* = 0.426, 0.152, and 0.184). These findings confirmed that the AH and control groups were demographically well matched, minimizing potential confounding in later analyses.

**Table 1:** Sociodemographic characteristics of the study population.

		Age3–5			Age5–8			Age8–11		
Group		Control (*n* = 30)	AH (*n* = 30)	p	Control (*n* = 30)	AH (*n* = 30)	p	Control (*n* = 30)	AH (*n* = 30)	p
Age (years)		4.3 ± 0.4	4.1 ± 0.3	0.108	7.1 ± 0.3	6.9 ± 0.5	0.065	9.8 ± 1.4	10.3 ± 0.7	0.086
Gender	Girl	17 (56.7 %)	14 (46.7 %)	0.438	13 (43.3 %)	16 (53.3 %)	0.438	15 (50 %)	18 (60 %)	0.436
	Boy	13 (43.3 %)	16 (53.3 %)		17 (56.7 %)	14 (46.7 %)		15 (50 %)	12 (40 %)	
BMI (kg/$m^{2}$)		18.67±3.47	18.31±2.84	0.662	17.54±4.24	18.08±2.80	0.563	19.35±3.89	18.96±5.23	0.744
Parents' education level	Illiterate	2 (6.7 %)	3 (10 %)	0.757	5 (16.7 %)	3 (10 %)	0.235	1 (3.3 %)	2 (6.7 %)	0.190
	Primary level	7 (23.3 %)	10 (33.3 %)		5 (16.7 %)	12 (40 %)		9 (30 %)	12 (40 %)	
	Secondary level	13 (43.3 %)	11 (36.7 %)		12 (40 %)	10 (33.3 %)		16 (53.3 %)	8 (26.7 %)	
	Graduate and above	8 (26.7 %)	6 (20 %)		8 (26.7 %)	5 (16.7 %)		4 (13.3 %)	8 (26.7 %)	
Area of residence	Rural	10 (33.3 %)	13 (43.3 %)	0.426	6 (20 %)	11 (36.7 %)	0.152	14 (46.7 %)	9 (30 %)	0.184
	Urban	20 (66.7 %)	17 (56.7 %)		24 (80 %)	19 (63.3 %)		16 (53.3 %)	21 (70 %)	

Data are expressed as mean±SD or n ( %), * *p* < 0.05. BMI, body mass index.

### Age-Dependent alterations in sagittal jaw relationships associated with adenoid hypertrophy

**Fig. 3:**
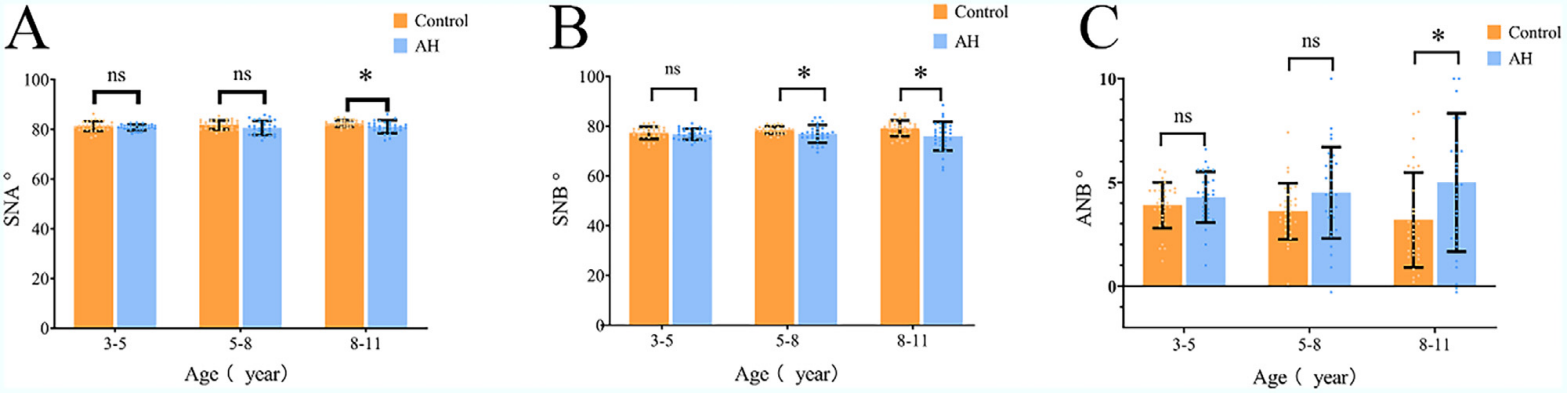
SNA(A), SNB(B), and ANB(C) among the study population. Data are expressed as mean±SD, * *p* < 0.05.

To evaluate age-related sagittal jaw changes associated with AH, SNA, SNB, and ANB angles were compared across three age groups: 3–5, 5–8, and 8–11 years (Figure 3A-C). No significant differences were observed between groups at 3–5 years. At 5–8 years, the AH group showed significantly lower SNB (*p* < 0.05) with unchanged SNA, while ANB showed a nonsignificant upward trend. By 8–11 years, both SNA and SNB were significantly reduced in the AH group (*p* < 0.05), with a larger reduction in SNB resulting in significantly increased ANB. These findings indicated that mandibular retrusion progressively worsened with age, contributing to increased sagittal discrepancies in children with AH.

### Progressive alterations in dentoalveolar position and occlusal plane angulation in children with adenoid hypertrophy

**Fig. 4:**
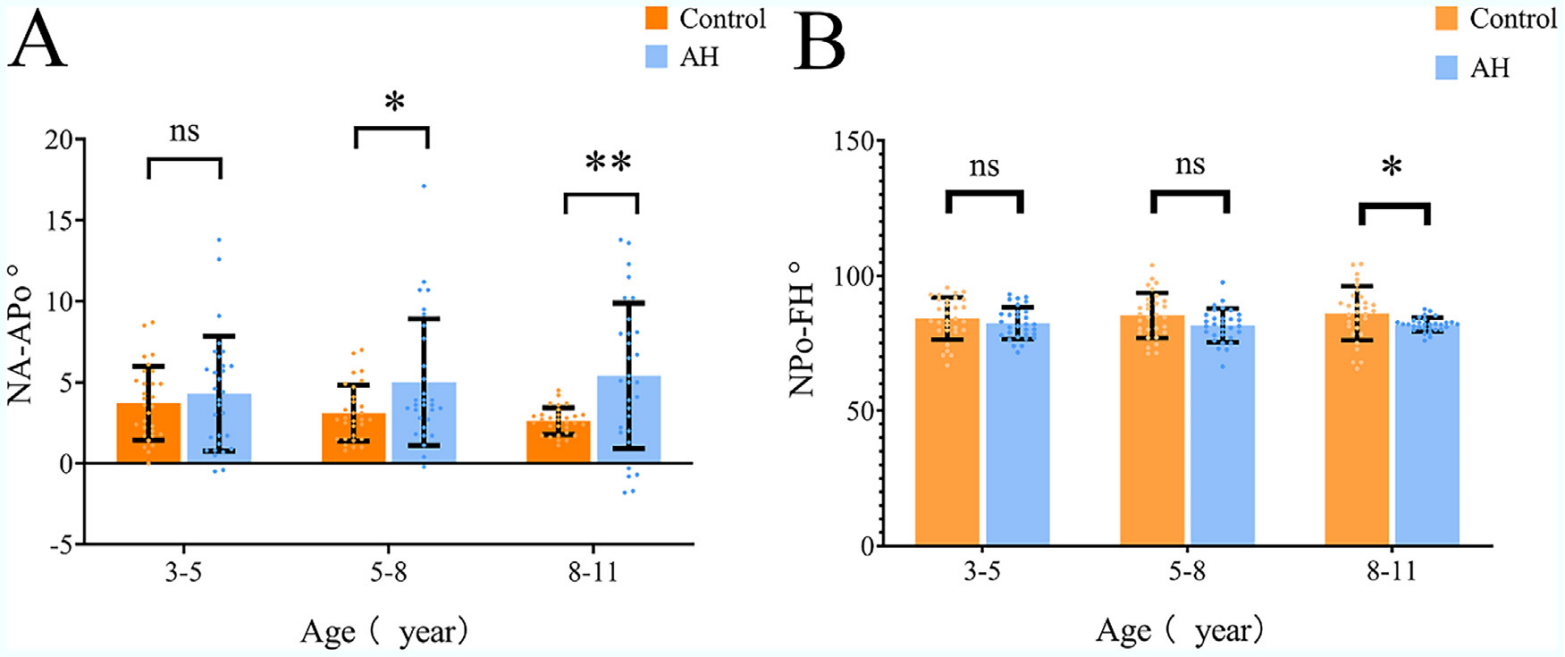
NA-Apo(A), NPo-FH(B) among the study population. Data are expressed as mean ± SD, * *p* < 0.05.

To assess age-related changes in dentoalveolar position and occlusal plane orientation associated with AH, NA-Apo and NPo-FH angles were compared across age groups (Figure 4A-B). No significant differences were found at 3–5 years. From 5–8 years onward, the NA-Apo angle was significantly higher in the AH group, becoming more pronounced at 8–11 years (*p* < 0.05, *p* < 0.01), indicating increased dentoalveolar protrusion. Similarly, the NPo-FH angle significantly decreased in the AH group at 8–11 years (*p* < 0.05), showing posterior tilting of the occlusal plane. Thus, AH was associated with progressive age-related changes in anterior tooth position and occlusal plane angulation.

### Age-Related increase in overjet without skeletal length changes in children with adenoid hypertrophy

To assess skeletal and dental changes associated with AH, linear measurements of ANS-Ptm (maxillary length), Co-Gn (mandibular length), and overjet were compared across three age groups (Table 2). ANS-Ptm and Co-Gn values showed no significant differences between the AH and control groups at any age, indicating that maxillomandibular skeletal lengths were not substantially affected by AH. However, overjet values increased progressively with age in the AH group. While no significant difference was observed in the 3–5 year group, overjet was significantly greater in the AH group at 5–8 years (*p* = 0.047) and 8–11 years (*p* = 0.007) compared to controls. The mean overjet difference (AH–control) was 1.8 mm at 5–8 years (95 % CI: 0.02–3.58; Cohen’s *d* = 0.52) and 2.8 mm at 8–11 years (95 % CI: 0.80–4.80; Cohen’s *d* = 0.72), indicating moderate effect sizes. These results suggest that jaw lengths were unchanged, but AH was linked to an age-related increase in overjet, likely due to mandibular retrusion and dentoalveolar compensation.

**Table 2:** ANS-Ptm, Co-Gn, Overjet values among the study population.

Group	ANS-Ptm ($\bar{x}$±*s*, mm)			Co-Gn ($\bar{x}$±*s*, mm)			Overjet ($\bar{x}$±*s*, mm)		
Age (years)	3–5	5–8	8–11	3–5	5–8	8–11	3–5	5–8	8–11
Control	40.3 ± 1.71	43.5 ± 4.473	44.6 ± 2.478	93.6 ± 10.487	97.3 ± 16.741	102.4 ± 13.399	2.9 ± 2.608	3.1 ± 3.086	2.8 ± 3.23
AH	40.6 ± 1.928	42.2 ± 5.929	42.9 ± 4.909	94.1 ± 14.576	97.2 ± 16.308	101.8 ± 12.375	3.3 ± 2.598	4.9 ± 3.762	5.6 ± 4.406
p	0.526	0.342	0.096	0.879	0.981	0.858	0.554	0.047	0.007

Data are expressed as mean±SD, * *p* < 0.05.

## Discussion

AH is commonly associated with upper airway obstruction and mouth breathing, which may influence craniofacial development. During early childhood (3–5 years), skeletal morphology remains largely unchanged, owing to the inherent plasticity of maxillomandibular growth and the compensatory mechanisms of the deciduous dentition phase [6,9]. In children aged 5 to 8 years and 8 to 11 years with AH, SNB angles are significantly lower, indicating mandibular retrusion. ANB angles are significantly higher, reflecting maxillary protrusion or mandibular retrusion. These findings are consistent with a progressive skeletal Class II pattern [6,7]. Altered breathing patterns and oral posture have been proposed to affect craniofacial growth direction and jaw posture.(1,4) Multivariate regression indicates that the AH/Np ratio correlates significantly with SNA and SNB changes, with snoring duration and mouth breathing serving as primary drivers of craniofacial morphological alterations [9]. Previous studies also link AH to sagittal skeletal discrepancies, particularly mandibular retrusion and altered craniofacial proportions [6]. Overall, these findings highlight early mixed dentition as a key window for monitoring and interdisciplinary evaluation [4].

Our age-stratified analyses revealed a stage-dependent pattern. No measurable skeletal or dentoalveolar differences were detected at 3–5 years. Early mixed dentition (5–8 years) was characterized by emerging mandibular retrusion and dentoalveolar compensation (SNB↓, NA-Apo↑). By 8–11 years, a broader deterioration was observed, with reductions in both SNA and SNB, an increased ANB, posterior occlusal plane inclination, and greater overjet. This within-cohort gradient may reflect an age- and duration-related worsening of AH-associated dentofacial alterations, rather than a uniform phenotype across childhood.

Craniofacial adaptations induced by AH extend to the dentoalveolar structures. In both the 5–8 and 8–11 year age groups, children with AH exhibited significantly increased NA-Apo angles, indicative of upper incisor proclination, a compensatory mechanism for mandibular retrusion [9]. In the 8–11 year cohort, the NPo-FH angle was significantly reduced, indicating posterior occlusal plane inclination, which may be related to tongue depression and altered masticatory force distribution from mouth breathing [6,7]. An open-mouth posture upsets perioral muscle balance, reducing lip pressure on the upper incisors and increasing tongue thrust, which worsens incisor proclination. Additionally, abnormal mandibular positioning alters occlusal loading patterns, ultimately influencing alveolar bone morphology [1,11]. AH patients consistently exhibit anterior alveolar protrusion alongside increased mandibular plane angles (MP/SN), highlighting concurrent sagittal and vertical discrepancies [6]. Early orthodontic assessment should identify these dentoalveolar compensations, and myofunctional therapy (e.g., lip-seal training) may help slow malocclusion progression.

Although maxillomandibular lengths (ANS-Ptm, Co-Gn) remain unchanged in children with AH, overjet increases steadily after age 5, peaking in the 8–11-year group [7,9]. These changes reflect mandibular retrusion and compensatory upper incisor proclination rather than skeletal hypoplasia, together worsening horizontal dental discrepancies [12]. Previous studies have demonstrated significantly higher rates of deep overjet in AH populations compared to controls (*p* < 0.05), with overjet positively correlated with maxillary protrusion and incisor inclination [13,14]. Furthermore, the prevalence of AH among patients with skeletal Class II malocclusion has been reported as high as 80 %, supporting overjet as an early clinical indicator of AH-related craniofacial abnormalities [15,16]. Overjet assessment should be incorporated into AH screening protocols. In patients presenting with Class II malocclusion, airway obstruction should be carefully evaluated [12,15]. Importantly, in our cohort the mean overjet increased to 4.9 mm (5–8 years) and 5.6 mm (8–11 years) in the AH group, compared with 3.1 mm and 2.8 mm in controls, supporting that the observed differences are not only statistically significant but also clinically noticeable.

Consistent with this stage-dependent progression, previous work suggests that symptom duration is a key predictor of dentofacial changes [9]. Age-specific analyses show that vertical discrepancies peak at 6–9 years, while sagittal discrepancies intensify at 9–12 years [17]. Postpubertal cases often exhibit a shift in OSA etiology from AH to obesity [18]. During the deciduous phase, nasal patency should be optimized, whereas in the mixed dentition phase, airway management should be combined with functional growth-modulating appliances [19].

Our findings are consistent with age-stratified reports showing that AH is associated with sagittal skeletal disharmony, driven mainly by mandibular retrusion and an increased ANB, with the signal most evident during the mixed-dentition stages [6,7]. The age-related worsening observed in our cohort is also in line with evidence that longer symptom duration (for example, habitual snoring and chronic mouth breathing) is associated with larger shifts in SNA and SNB [9]. Notably, although mandibular size or length differences have been described in some age windows, no group differences were detected for ANS-Ptm or Co-Gn in the present sample. This pattern suggests that, in prepubertal children, AH may preferentially influence jaw position and rotational pattern, accompanied by dentoalveolar compensation, rather than producing a primary deficiency in skeletal length. Our restriction to prepubertal skeletal maturity (CS1-CS2) and the use of ANS-Ptm/Co-Gn as length surrogates may also partly explain why skeletal length differences observed in other cohorts were not detected here. Between-study differences may reflect variation in age ranges, diagnostic definitions (isolated AH vs. adenotonsillar hypertrophy), population background, and whether vertical growth measures were included.

Dentoalveolar compensation varies by skeletal class. In Class I, vertical alveolar adaptation fine-tunes incisor inclination to preserve occlusion [20,21]. In Class II, early use of functional appliances before puberty limits maxillary growth and enhances mandibular compensation, improving alignment and stability [22,23]. Children with AH often exhibit increased mandibular plane angle (MP/SN) and alveolar height, leading to an elongated facial profile and more severe malocclusion [7,13]. AH also reshapes the airway and displaces the hyoid bone posteriorly [24,25], elevating obstructive sleep apnea risk in older children, especially when obesity and mandibular anatomy (height, SNB) are contributory [18,26].

Lateral cephalometry is the standard for AH diagnosis, though accuracy depends on obstruction severity [27]. Effective management requires collaboration between ENT specialists, pediatric dentists, and orthodontists to ensure early intervention and reduce malocclusion risk. Adenoidectomy or other surgical treatments may be indicated [28,29]. Tonsillar hypertrophy often coexists, further reducing bite force and airway volume [28], but dentoalveolar compensation can partly mitigate skeletal imbalances [30,31]. The strong association between AH, mouth breathing, and facial growth highlights the necessity of personalized treatment planning.

Clinically, the age-stratified analyses indicate that AH-associated dentofacial deviations become evident from early mixed dentition (5–8 years) and appear to progress by 8–11 years. In particular, overjet increased from 3.1 mm (controls) to 4.9 mm (AH) at 5–8 years and from 2.8 mm to 5.6 mm at 8–11 years (Table 2), corresponding to mean differences of 1.8–2.8 mm with moderate effect sizes. Increased overjet and NA-Apo, together with a reduced SNB angle, may provide pragmatic screening cues that warrant targeted upper-airway evaluation. Since jaw lengths (ANS-Ptm and Co-Gn) did not differ between groups, the craniofacial effects likely reflect positional changes and dentoalveolar compensation rather than true skeletal length deficiency. These findings support early airway management and coordinated interceptive orthodontic and myofunctional intervention during the mixed-dentition period. Children with persistent mouth breathing or snoring who develop a Class II trend (e.g., reduced SNB with overjet rising to ∼5–6 mm) may benefit from earlier ENT–orthodontic referral, especially at 5–8 years when changes first appear.

This study still has several limitations. First, the retrospective design precludes drawing causal links between adenoid hypertrophy and craniofacial changes. Second, the use of two-dimensional lateral cephalograms may introduce measurement inaccuracies due to projection and landmark identification errors; the lack of 3D imaging restricts the precision of anatomical evaluation. Third, the absence of longitudinal follow-up in treated cases precludes evaluation of treatment effects on craniofacial development. Fourth, selection bias is possible because AH cases came from a clinical database, whereas controls were recruited from a health examination center. Fifth, despite matching for age, sex, and BMI and restricting analyses to CS1-CS2, unmeasured confounding may remain. Sixth, because multiple outcomes were tested across age groups, the findings are exploratory and need confirmation in prospective studies. Thus, prospective longitudinal studies using advanced imaging techniques are warranted to validate these findings.

Future work should prioritize (i) prospective longitudinal follow-up across dentition stages to define developmental trajectories and improve causal inference; (ii) pre–post intervention analyses (for example, after medical therapy/adenoidectomy and/or myofunctional or functional orthopedic-orthodontic treatment) to assess reversibility of positional and dentoalveolar changes; (iii) integration of objective airway and sleep measures with cephalometric phenotyping; and (iv) selective use of CBCT to refine anatomic assessment while limiting radiation exposure.

## Conclusion

In this retrospective, age-stratified analysis, adenoid hypertrophy was associated with age-dependent mandibular retrusion, dentoalveolar protrusion, posterior occlusal plane inclination and increased overjet, whereas maxillary and mandibular lengths (ANS-Ptm and Co-Gn) did not differ between groups. These results suggest that, in prepubertal children, AH is linked mainly to jaw position/rotation and dentoalveolar compensation rather than skeletal length deficiency. Early recognition of airway obstruction and interdisciplinary evaluation during mixed dentition may help limit progression of dentofacial discrepancies, but prospective longitudinal studies are required to confirm causality and treatment effects.

## Statements of ethics

The study was approved by Children’s Hospital of Nanjing Medical University (#202405006–1). The study was performed in strict accordance with the Declaration of Helsinki, Ethical Principles for Medical Research Involving Human Subjects.

## Informed consent

All patients signed the informed written consent.

## Funding

None.

## Consent for publication

Not applicable.

## Data Availability Statement

The data that support the findings of this study are available from Jian-Ya Zhang upon request.

## CRediT authorship contribution statement

**Xue-Ru Zhang:** Data curation, Formal analysis, Funding acquisition, Validation, Supervision, Writing – original draft. **Jian-Ya Zhang:** Data curation, Formal analysis, Funding acquisition, Validation, Supervision, Writing – original draft.

## Conflicts of interest

The authors declare no conflicts of interest.
